# Optimized protocol for DNA/RNA co-extraction from adults of *Dirofilaria immitis*

**DOI:** 10.1016/j.mex.2019.10.023

**Published:** 2019-10-31

**Authors:** Chiara Lucchetti, Marco Genchi, Luigi Venco, Chiara Bazzocchi, Laura H. Kramer, Alice Vismarra

**Affiliations:** a Department of Veterinary Science, University of Parma, Parasitology Unit, Parma, 43126, PR, Italy; b Clinica Veterinaria Lago Maggiore, Arona, 28041, NO, Italy; c Department of Veterinary Science, University of Milan, Milan, 20133, MI, Italy

**Keywords:** *DNA/RNA co-extraction from a single adult worm of D. immitis*, TRIzol®, RNA extraction, DNA extraction, Adult worms

## Abstract

*Dirofilaria immitis*, the etiologic agent of canine heartworm disease, like several other filarial nematodes, harbors the bacterial endosymbiont *Wolbachia*. To investigate metabolic and functional pathways of *D. immitis* and *Wolbachia* individually, along with their interactions, the use of both transcriptomic and genome analysis has becoming increasingly popular. Although several commercial kits are available for the single extraction of either DNA or RNA, no specific protocol has been described for simultaneous extraction of DNA and RNA from such a large organism like an adult *D. immitis*, where female worms generally reach ∼25 cm in length. More importantly, adult worms of *D. immitis* can only be obtained either through necropsy of experimentally infected dogs or by minimally-invasive surgical heartworm removal of naturally infected dogs. This makes each individual worm sample extremely important. Thus, in the context of a project aimed at the evaluation of both gene expression analysis and *Wolbachia* population assessment following different treatments, an optimized protocol for co-extraction of DNA and RNA from a single sample of adult *D. immitis* has been developed.

• An optimized method for DNA/RNA co-extraction from large size nematodes using TRIzol® reagent.
• Allows maximum exploitation of unique samples as adults of *D. immitis*.

## Graphical abstract

**Figure:**
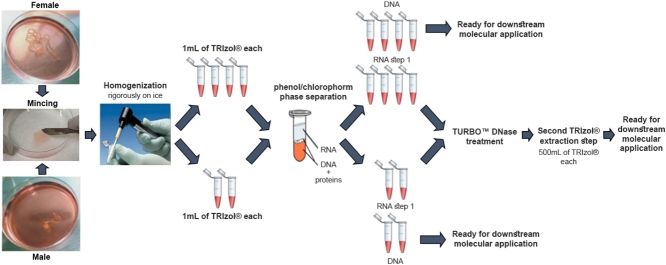

**Specification Table**

**Table:** 

Subject Area:	*Veterinary Science and Veterinary Medicine*
More specific subject area:	*Veterinary Parasitology*
Method name:	*DNA/RNA co-extraction from a single adult worm of D. immitis*
Name and reference of original method:	TRIzol® Reagent protocol. *Chomczynski, P. A reagent for the single-step simultaneous isolation of RNA, DNA and proteins from cell and tissue samples. BioTechniques 15 (3) (1993) 532-537* [1].
Resource availability:	https://www.thermofisher.com/order/catalog/product/15596026

## Method details

A method for co-extraction of DNA and RNA from a single worm of *D. immitis* has been validated using TRIzol® as the main reagent. The described extraction procedure grants maximum exploitation of a single sample of adult *D. immitis* worm, allowing the possibility of focusing on different levels, either on the parasite itself or on its symbiont, and thus investigating different aspects of their metabolisms.

### Preparation of *D. mmitis* adult worms

Following owners’ consent, 12 adult worms of *D. immitis*, respectively 6 females and 6 males, were collected during minimally-invasive surgical heartworm removal from two dogs previously diagnosed with patent *D. immitis* infection. Extracted worms were washed in Hank’s Balanced Salt Solution medium (Sigma-Aldrich®, Missouri, USA) at room temperature, checked for viability, counted and sexed. Three female and three male worms were individually placed into 15 mL Falcon tubes and stored at −80 °C. Single worms were used as starting material for the RNA/DNA co-extraction.

### Sample homogenization

Because female *D. immitis* are longer than males (30 cm vs. 20 cm, approximately), with approximately double the starting weight compared to males, the following steps of extraction were performed slightly differently between the two sexes.

Each frozen worm was homogenized individually. The worm was placed into a precooled Petri dish with 500 μL of cold TRIzol® Reagent (Ambion®, Foster City, USA). With the help of a sterile scalpel, the worm was chopped into tiny pieces, thin enough so that they could be picked easily up by trimmed 1000 μL tips, and then placed it into a sterile RNase and DNase free 1.5 mL Eppendorf tube. Each worm was homogenized using an electric pellet pestle (Sigma-Aldrich®, Missouri, USA) for 3 min. Then, an additional 500 μL of TRIzol® Reagent were added to partially homogenized sample, and after mixing it thoroughly, 500 μL of it were transferred into a new sterile tube. Homogenization with the electric pestle was repeated once more per each of the two tubes, for 2 min. For females, since their initial weight was twice that of males (∼220 mg and ∼115 mg respectively), the homogenization step was repeated one more time per each of the two samples just prepared. A further 500 μL of TRIzol® Reagent was added to each of the two tubes and, after mixing the sample thoroughly, 500 μL of sample were transferred into a new tube. Each sample was homogenized again for a 1 min. All steps were performed on ice, to avoid RNA degradation.

### DNA and RNA co-extraction

To each tube containing 500 μL of homogenized sample a further 500 μL of TRIzol® reagent were added. They were treated according the TRIzol® Reagent manufacturer protocols [1]. Once the three phases were separated and the aqueous phase set apart from the other two, the two isolation steps were performed once again according to the manufacturer protocol, although a few changes were made at the elution step for both RNA and DNA procedures.

The final elution of RNA samples was performed in 100 μL of DEPC water, and the rehydration of the sample was performed at 56 °C per 13 min. Some samples, due to the presence of a greater pellet, were placed in a water bath at 56 °C for a longer time, until the pellet was fully rehydrated.

The final elution of DNA samples was performed in 300 μL of DEPC water, and they were then placed in a water bath at 46 °C for 15 min.

After resuspension, all samples were briefly vortexed and analyzed at the spectrophotometer (BioSpectrometer® fluorescence with μCuvetta® G 1.0, Eppendorf, Hamburg, Germany). For each sample, data concerning concentration (ng/μL) and quality, described as 260/280 and 260/230 values were collected.

### RNA purification for down-stream molecular applications

In order to guarantee RNA quality which would allow down-stream analysis, such as quantitative/relative RT-PCR, each sample required the elimination of any possible residue of genomic DNA.

Therefore, a solution of 150 ng/μL of RNA in a final volume of 50 μL was prepared for each sample. To each sample, 5 μL of TURBO™ DNase buffer and 2 μL of TURBO™ DNase (Thermo Fisher Scientific, Waltham, USA) were added, followed by incubation at 37 °C for 30 min. After the first incubation, a further 2 μL of TURBO™ DNase enzyme was added and incubated again at 37 °C for other 30 min.

A 500 μL of TRIzol® Reagent was added to each tube and a second RNA extraction was performed following the manufacturer protocol. The final elution was performed in 35 μL of DEPC water and as performed for the first RNA extraction, pellets were rehydrated at 56 °C for 13 min. All samples were quantified at the spectrophotometer. Results obtained from both the first and the second RNA extraction as well as those obtained for DNA have been reported in Table1.

**Table 1:** Data collection reporting concentrations (ng/μL) as well as the 260/280 and 260/230 parameters measure by spectrophotometer analysis of three biological replicates per each sex. All samples were analyzed individually and per each of the six worms the mean values of the technical replicates were calculated and listed in the table. In the table are reported values for the overall mean and standard error of mean (SEM).

SEX	DNA			RNA STEP 1			RNA FINAL		
	ng/μL	260/280	260/230	ng/μL	260/280	260/230	ng/μL	260/280	260/230
**Female**									
1	137.65	1.31	0.88	358.63	1.81	0.82	152.95	1.84	0.70
2	212.83	1.32	0.91	354.00	1.82	0.79	181.55	1.95	1.19
3	201.18	1.31	0.74	512.78	1.72	0.57	161.38	1.83	1.15
MEAN	183.88	1.31	0.84	408.47	1.78	0.73	165.29	1.88	1.01
SEM	±35.10	±0.00	±0.09	±41.48	±0.02	±0.06	±18.65	±0.05	±0.15

**Male**									
1	55.45	1.37	0.61	261.80	1.75	0.37	160.45	1.86	1.03
2	29.60	1.36	0.39	323.25	1.83	1.07	168.10	1.86	1.46
3	10.30	1.75	0.17	304.10	1.87	1.25	166.60	1.80	1.68
MEAN	31.8	1.5	0.4	261.8	1.8	0.4	160.5	1.9	1.0
SEM	±14.59	±0.11	±0.14	±14.07	±0.02	±0.17	±2.24	±0.03	±0.14

### cDNA preparation and purity check

To obtain a fully pure RNA sample to be used for gene expression analysis, an additional purification step was performed. RNA samples were treated with AccuRT Genomic DNA Removal Kit (Abm, Richmond, Canada) according to the manufacturer protocol. Starting with 300 ng of final RNA, and followed by an incubation at room temperature of 8 min. For cDNA synthesis, the OneScript® cDNA Synthesis Kit (Abm, Richmond, Canada) was used. The presence of any genomic contamination was checked by PCR reaction using GOTAQ Flexi DNA Polymerase Green G2 (Fisher MB, Rome, Italy) and 0.35 μM of specific primers designed on Dim-pgp-10 for cDNA (DimmScaf48-cDNA-F8: 5′-GCCATCGTAGGTCCATCAGGTTCTGGT-3′; DimmScaf48-cDNA-R12: 5′-TGTTCAACTGAAACGACCACACGTC-3′) (Sigma-Aldrich®, Missouri, USA) [2]. The amplification protocol was characterized by a denaturation step at 95 °C for 1,30 min, followed by 40 repeated cycles: 95 °C for 15 s; 59.4 °C for 1 min with a final elongation of 72 °C for 5 min. Amplicons were run on a 2% agarose gel. For pure cDNA only one band would be of 155bp while in presence of genomic DNA contamination a second band of amplification would be visible at 476bp.

### RNA extraction through Phasemaker™ Tubes

To validate the efficiency of proposed protocol, RNA products obtained with the just described protocol were compared to those obtained with the use of a product specific for the optimization and simplification of RNA isolation: Phasemaker™ Tubes (Thermo Fisher Scientific, Waltham, USA).

Three adult worms of *D. immitis* per each sex were analyzed. Thus, two samples per each female and one per each male were used for the extraction procedure with Phasemaker™ Tubes. The manufacturer’s protocol was followed. All remaining samples, respectively two samples per each female and one per each male, were processed using the proposed co-extraction protocol. Results from both RNA extractions are reported and compared in Table 2.

**Table 2:** Comparison between concentration and quality of samples from untreated worms (Control) extracted following either the newly developed protocol or using the Phasemaker™ Tubes manufacturer protocol. Mean ± SEM values calculated between the three biological samples are reported.

Samples		RNA step 1			RNA final		
		ng/μL	260/280	260/230	ng/μL	260/280	260/230
Female	Novel protocol	616.00 ± 44.67	1.90 ± 0.01	1.50 ± 0.10	141.52 ± 14.00	2.11 ± 0.13	1.57 ± 0.22
	Phasemaker™ Tubes protocol	859.52 ± 46.13	1.81 ± 0	1.31 ± 0.10	100.75 ± 10.77	2.16 ± 0.15	1.44 ± 0.44
Male	Novel protocol	320.87 ± 16.62	1.76 ± 0.06	0.98 ± 0.15	205.33 ± 12.42	1.75 ± 0.06	1.16 ± 0.36
	Phasemaker™ Tubes protocol	346.23 ± 43.81	1.66 ± 0.06	0.81 ± 0.10	167.50 ± 0.02	1.70 ± 0.02	0.81 ± 0.27

When compared, results obtain from the Phasemaker™ Tubes with those obtained following the co-extraction protocol; both their quality and quantity of RNA appeared similar between the protocols. In fact, no statistical difference was observed between the two different extraction procedures when the single worms were considered separately. On the other hand, when the overall results from each sex were compared, the statistical analysis showed a marked significant difference (p-value = 0.005) in female step 1 RNA and a less significant difference for final RNA (p-value = 0.03). No difference was observed for all RNA extracted from males. This difference is probably related to the difference in size among the female worms used for this assay.

Results obtained validate the possibility of using the proposed protocol as an efficient option for simultaneous extraction of good quality of both DNA and RNA from a unique sample of adult *D. immitis*. To the authors’ knowledge, this is the first attempt to develop a protocol for DNA/RNA co-extraction in nematodes.

## Additional information

Until now, co-extraction has never been described for nematodes and, to the authors’ knowledge, there are no reports of this procedure from a single adult nematode sample, with a large size such as that of *D. immitis*. Most of the existing methods for molecular analysis of filarial nematodes have focused on single RNA or DNA extraction. According to previous studies, DNA is generally isolated by means of either commercial kits such as DNeasy Blood and Tissue Kit (QIAGEN, Germany) and QIAamp® DNA Mini Kit (Qiagen, Venlo, The Netherlands), or of alternative protocols involving a combination lysis buffers, composed primarily of Tris-HCl and K proteinase enzyme [[3], [4], [5], [6]]. For RNA extraction, the use of commercial kit such as Micro Fast Track™ purification kit (Thermo Fisher Scientific, Waltham, USA) or RNeasy kit (Qiagen, Valencia, CA, USA), as well as the use of TRIzol® reagent following sample digestion in Tris-EDTA have been extensively described [[7], [8], [9]].

Thus, the purpose of this study was not to develop a novel protocol for ribonucleic acid co-extraction, but to adapt and optimize an already existing method for simultaneous DNA/RNA isolation from a single adult of *D. immitis*. This optimization grants full exploitation of a single sample, so that both molecules could be used for specific molecular analysis, such as quantitative RT-PCR, which otherwise would be impossible to perform on a same sample. In particular, this new protocol will allow to correlate genes expression by the parasite with bacterial load; hence it may offer new insights into the effect of *Wolbachia* reduction on the parasite host’s biology and function, and thus into the adulticide effects of specific treatments.

## Declaration of Competing Interest

The authors declare that they have no known competing financial interests or personal relationships that could have appeared to influence the work reported in this paper.
